# *EML4-ALK*与*EGFR*基因突变共存型非小细胞肺癌研究进展

**DOI:** 10.3779/j.issn.1009-3419.2011.11.09

**Published:** 2011-11-20

**Authors:** 珠 曾, 一龙 吴

**Affiliations:** 510080 广州，广东省肺癌研究所，广东省人民医院，广东省医学科学院 Guangdong Lung Cancer Institute, Guangdong General Hospital & Guangdong Academy of Medical Sciences, Guangzhou 510080, China

**Keywords:** 肺肿瘤, *ELM4-ALK*融合基因, 表皮生长因子受体, Lung neoplasms, Human *EML4-ALK* fusion protein, Epidermal growth factor receptor (EGFR)

## Abstract

肺癌是最常见的恶性肿瘤之一，其中非小细胞肺癌（non-small cell lung cancer, NSCLC）占肺癌的80%-85%。分子靶向治疗是目前NSCLC最热门也是最具前景的领域之一，其中的热点分子包括表皮生长因子受体（epidermal growth factor receptor, EGFR）、棘皮动物微管样蛋白4-间变淋巴瘤激酶（echinoderm microtubule associated protein like4-anaplastic lymphoma kinase, EML4-ALK）等。既往研究认为*EML4-ALK*融合基因与*EGFR*突变不能共存。近期陆续报道了*EML4-ALK*融合基因与*EGFR*突变共存的病例。本文就*EML4-ALK*融合基因及*EGFR*突变基因的分子结构、发生率和目前已报道双突变患者的临床特点等进行综述。

## Abstract (en)

Lung cancer is one of the most prevalence malignances, of which non-small cell lung cancer (NSCLC) account for 80%-85% of lung cancer. Molecular target therapy is one of the most popular and promising field of NSCLC treatment, and its hotspots includes EGFR (epidermal growth factor receptor), EML4-ALK (echinoderm microtubule associated protein like4-anaplastic lymphoma kinase), *etc.* Former researches indicated that *EML4-ALK* fusion and *EGFR* mutation were excluded mutually. However, cases of patients harbored concomitant *EML4-ALK* fusion gene and *EGFR* mutation have been reported continuously at recent. This review aims to summarize the incidence and molecular structure of *EML4-ALK* fusion gene and *EGFR* mutation, as well as clinical features of patients with the concomitant genes induced NSCLC.

肺癌是美国病死率最高的恶性肿瘤^[1]^。非小细胞肺癌（non-small cell lung cancer, NSCLC）占肺癌的80%-85%，其传统治疗方法包括化疗、放疗、手术等。随着肿瘤发病机制研究的不断深入，分子靶向治疗已经成为了研发热点，并初步取得了疗效。表皮生长因子受体（epidermal growth factor receptor, EGFR）、棘皮动物微管样蛋白4-间变淋巴瘤激酶（echinoderm microtubule associated protein like4-anaplastic lymphoma kinase, EML4-ALK）等靶点的分子抑制剂在临床上的成功让学者们将更多的精力投入到分子靶向的研究中。2011年美国临床肿瘤学会（American Society of Clinical Oncology, ASCO）大会提示肿瘤诊疗已经进入分子靶向治疗的时代。既往研究^[2-4]^认为NSCLC中*EML4-ALK*融合基因与EGFR及KRAS（Kirsten Ras）突变是相互排斥的。近期陆续发现有*EML4-ALK*融合基因和*EGFR*突变共存的案例。在强调个体化治疗的时代，这部分患者的临床表现特点和治疗方法值得研究，本文就这些问题进行综述。

## 1 *EML4-ALK*融合基因的分子结构和在NSCLC中的发生率

间变淋巴瘤激酶（anaplastic lymphoma kinase, ALK）是一个由细胞外配体结合区、跨膜区及胞内的酪氨酸激酶区组成的1, 620个氨基酸的跨膜蛋白，属于胰岛素受体家族^[5]^。

棘皮动物微管相关样蛋白4（echinoderm microtubule-associated protein-like 4, EML4）属于棘皮动物微管相关样蛋白家族，由N末端碱基区（N-terminal basic region）、疏水的棘皮动物微管相关蛋白（hydrophobic echinoderm microtubule-associated protein-like protein, HELP）区以及WD重复区（WD-repeat region）3部分构成。WD重复区是一段通常以色氨酸-天冬氨酸结尾的氨基酸的序列，包含了4个-16个重复的单元，其所有的单元一起形成一个beta螺旋结构。WD重复区作为一个大家族广泛存在于真核生物中，负责细胞信号转导、细胞周期控制、细胞凋亡调控等。研究^[2]^证明在*EML4-ALK*融合基因中，以上3个部分都与肿瘤的形成有关，其中N末端碱基区的作用最为重要。

*EML4-ALK*融合基因是由日本学者Soda等在1名62岁有吸烟史的男性腺癌患者的肿瘤组织中首次发现的。基因的重排发生在2号染色体短臂上的2区1带和2区3带，由*ALK*基因的3′端与*EML4*基因的5′倒位融合形成。*EML4*基因断裂后形成不同长度的外显子拼接片段，插入位置相对保守的*ALK*基因的19号、20号外显子之间，从而形成有11个变体的EML4-ALK融合蛋白^[2, 3, 6-11]^。融合基因大多都有致瘤性^[12]^，其中变体1最常见，变体3a/3b次之^[13]^。以最常见的变体1为例，EML4在WD重复区处断裂，形成一个由N末端碱基区、HELP区、部分WD重复区组成的片段与ALK的胞内区融合^[2]^。融合基因中EML4的启动子位于ALK胞内酪氨酸激酶的上游，从而导致融合基因活化，表达EML4-ALK融合蛋白。通过EML4的胞外结构形成的二聚体使ALK在缺乏配体的情况下受体持续自磷酸化，进而持续激活下游细胞信号通路导致细胞恶性转化^[14]^。

文献^[15]^报道不加选择的NSCLC患者*EML4-ALK*融合基因总发生率为3.4%（范围0.4%-11.6%）。东方人群NSCLC患者的总发生率为4.1%，欧美人群为2.5%。Shaw等^[9]^在女性、亚裔、不吸烟或者是轻度吸烟（每年吸烟≤10包或戒烟≥1年者）这4个条件至少满足2个条件的141名肺腺癌患者的肿瘤组织中用荧光原位杂交技术（fluorescent in situ hybridization, FISH技术）检测了*EML4-ALK*融合基因的发生率，结果显示其发生率高达13%;在EGFR和KRAS均为野生型的不吸烟/轻度吸烟者则为33%。Zhang等^[4]^用RACE-coupled PCR sequencing法在中国NSCLC患者中进行检测，结果表明中国的非选择性NSCLC患者中该融合基因的发生率为11.6%，EGFR和KRAS均为野生型的不吸烟/轻度吸烟者高达42.8%。

## 2 *EGFR*突变基因的分子结构及发生率

EGFR具有受体酪氨酸激酶活性，属于Ⅰ型生长因子受体家族，由1个胞外配体结合区、跨膜区和1个胞内区构成。EGFR与配体结合后形成同源或异源二聚体，引起构象变化，激活下游的多个信号通路，在肿瘤发生与转移的多个环节中起调节作用。

*EGFR*基因位于7号染色体短臂的12-14区，由28个外显子组成，突变多位于19-21外显子（酪氨酸激酶功能区），其突变主要有3种类型：①19外显子碱基缺失，改变了受体ATP结合囊（ATP-binding pocket, ABP）角度，增强了肿瘤细胞对酪氨酸激酶抑制剂（tyrosine kinase inhibitors, TKIs）的敏感性^[16, 17]^，Shigematsu等^[18]^发现19外显子突变的发生率最高，占*EGFR*突变总数的50%以上; ②20外显子点突变或碱基插入突变，20外显子的点突变主要是第790位密码子出现C-T转换，从而引起该处的苏氨酸转变为甲硫氨酸（T790M），这一突变多见于药物治疗后的复发患者，突变后肿瘤细胞对TKIs产生抵抗^[19]^。插入突变出现在第770-775位密码子，插入一个长约3个-9个碱基的片段，约5%的NSCLC患者出现插入突变。体外实验表明，与19及21外显子突变相比，20外显子插入突变的患者对EGFR-TKIs不敏感并且大多数出现疾病进展^[20]^;③21外显子点突变，主要是851位密码子出现T-G转换，亮氨酸转变为精氨酸（L858R），突变提高了肿瘤细胞对TKIs的敏感性^[17]^。

在非选择性NSCLC患者中，EGFR在美国的NSCLC腺癌患者中突变率约为15%^[21]^;在亚裔人群为30%-50%，且大部分是腺癌和支气管肺泡癌^[22]^。

## 3 EGFR-TKIs、ALK-TKIs靶向治疗的疗效

ALK的口服抑制剂PF-02341066（Crizotinib）的Ⅰ期临床试验（NCT00585195或A8081001）开始于2006年。2009年ASCO年会上报道，PF-02341066的有效率达53%（10/19），疾病控制率达到79%（12/19）。鉴于良好的安全性和较高有效率，美国食品及药物管理局（Food and Drug Administration, FDA）批准此药直接进入Ⅲ期临床试验。目前该药的全球Ⅲ期临床试验正在进行中。2011年ASCO对Crizotinib的Ⅰ期临床试验结果进行了更新，入组NSCLC患者已达119例，中位无进展生存期达到10个月（95%CI: 8.2-14.7），客观有效率为61%（95%CI: 52%-70%）。中位有效应答时间为48 w，中位生存时间达1年的概率为81%^[23]^。

与EGFR-TKIs及其它酪氨酸激酶抑制剂一样，即使在治疗初期Crizotinib能取得明显的疗效，肿瘤最终也会对Crizotinib耐药。日本的研究者Choi等^[24]^在1例Crizotinib治疗后复发患者的标本中发现了两处新突变，即4374G→A和4493C→A。两个碱基突变分别导致了氨基酸C1156Y和L1196M的改变，从而影响ALK与Crizotinib或ATP的结合。Choi等推测，这两个点突变能分别独立导致耐药发生，其中管家基因L1196M突变导致的耐药性比C1156Y更强。

针对管家基因L1196M耐药后的治疗，Katayama等^[25]^提出可以用比Crizotinib活性更强的ALK抑制剂如TAE684及AP26113，或是HSP90抑制剂进行治疗。Sakamoto等^[26]^也用细胞实验证实了ALK抑制剂CH5424802能高选择性抑制L1196M突变的细胞生长。

EGFR抑制剂吉非替尼和厄洛替尼目前已经广泛应用于晚期NSCLC临床各线的治疗。IPASS研究^[27]^比较了一线使用EGFR-TKIs与一线使用化疗药物的疗效，发现EGFR突变患者是吉非替尼治疗的最大获益人群。鉴于IPASS及其他三项基于*EGFR*突变的临床试验研究结果，ASCO于今年4月发布了PCO（provisional clinical opinion），建议对晚期初治的NSCLC患者进行*EGFR*突变检测以决定患者是进行EGFR-TKIs治疗还是进行化疗^[21]^。

## 4 *EML4-ALK*与*EGFR*突变共存患者的临床特征

*EML4-ALK*融合基因型患者与*EGFR*基因突变型患者有相似的临床特征^[9, 28]^，如多见于不/轻度吸烟的腺癌患者，但*EML4-ALK*融合基因型患者多为年轻的男性患者，且不能从EGFR-TKIs治疗中获益^[9]^。

既往研究^[2, 6, 7, 9, 13]^认为*EML4-ALK*融合型基因与*EGFR*基因突变是互为独立的分子事件，*EML4-ALK*融合型基因可能是继KRAS之后的EGFR对TKI耐药的另外一个原因。目前已报道了8例^[4, 6, 29-31]^*EML4-ALK*融合型基因与*EGFR*基因突变共存型患者（表 1，其中1例无临床信息未在表中列出），其所表现的临床特点与既往的研究有所不同。

**1:** 7例*EML4-ALK*融合基因和*EGFR*突变共存患者的临床特征 he clinical features of 8 patients with *EML4-ALK* fusion gene and *EGFR* mutation

Patient No.	1^[31]^	2^[31]^	3^[31]^	4^[31]^	5^[4]^	6^[29]^	7^[30]^
Ethnicity	Chinese	Chinese	Chinese	Chinese	Chinese	Chinese	Caucasian
Age (years)	44	56	50	70	56	72	48
Gender	Female	Female	Male	Male	Female	Female	Male
Smoking history	Never smoker	Never smoker	45 pack years	Never smoker	Never smoker	Never smoker	Never smoker
Histology	Adenocarcinoma	Adenocarcinoma	Adenocarcinoma	Adenocarcinoma	Adenocarcinoma	Adenocarcinoma	Adeno-squamous carcinoma
Primary lesion	The left upper lung lobe	The right upper lung lobe	The right upper lung lobe	The right upper lung lobe	The right upper lung lobe	The right upper lung lobe	The right upper lung lobe
TNM Staging	cT2aN3M1b（the left ribs and the thoracic vertebral bodies）Stage Ⅳ	cT4N3M1b (the brain) Stage Ⅳ	cT3N0M1a (pleura) Stage Ⅳ	cT1bN3M1b (the brain) Stage Ⅳ	T1N0M0 StageⅠa	Stage Ⅳ brain bone metastasis	cT1N0M1 (left tenth rib)
EGFR mutation status	Exon19 deletion	Exon21 L585	Exon21 L585	Exon21 L585	Exon19 deletion	Exon19 deletion	Exon19 deletion
EML4-ALK fuison	Variant 6	Variant 1	Variant 1	Variant 1	Variant 1	Variant 1	*
First line treatment	Gefitinib	Gefitinib	Sequential Gemcitabine/ Carboplatin+Erlotinib	Erlotinib	Surgery	Gefitinib	Cisplatin+Gemcitabine 6 cycles
First line treatment assessment	Partial response (122 days after treatment)	Partial response (36 days after treatment)	Partial response (8 weeks after treatment)	Partial response (105 days after treatment)	R0 resection#	Partial response	Partial response
The case Koivunen reported did not mention patient’s information in the paper, we did not present in the table. ^*^No report in the author’s article. ^#^Complete resection with no microscopic residual tumor.							

这7例大部分为女性（4/7）、亚裔（6/7）、无吸烟史（6/7）的晚期患者，中位年龄56岁，病理类型几乎都是腺癌（6/7），*EGFR*突变既可以是19外显子缺失（4/7）也可以是21外显子点突变（3/7），但EML4-ALK主要是变体1的融合。特别指出的是，在一线使用EGFR- TKIs的5例患者中，疗效均达到部分缓解（第7例患者二线治疗行右上肺切除+淋巴结清扫+肋骨切除术，三线治疗使用2个月厄洛替尼后死于疾病进展），这与传统认为的*EML4-ALK*融合基因的患者对EGFR-TKIs耐药的观点^[9]^显然不一致。

## 5 展望

*EML4-ALK*融合基因是当前研究的热点，极有可能像*EGFR*突变那样成为NSCLC的一个亚型。PF-02341066（Crizotinib）的Ⅲ期临床试验正在进行，Crizotinib能否像EGFR-TKIs一样成为NSCLC治疗上的一个里程碑值得期待。EGFR-TKIs目前已经广泛应用于临床的治疗，在治疗晚期*EGFR*突变的NSCLC患者中疗效明显。但对于*EML4-ALK*融合基因与*EGFR*基因突变共存型患者，目前的研究和报道还很少，仍有许多问题值得思考和研究，包括共存型患者其EML4-ALK与EGFR下游的分子生物学行为、案例报道中患者对EGFR TKIs的反应不一致的原因是否为EML4-ALK不同变体对EGFR TKIs的敏感性不同、仅第7例患者对EGFR-TKIs不敏感是否是化疗改变了肿瘤对TKIs的敏感性所致、对于共存型患者该如何应用TKIs、多靶点抑制剂会不会是共存型患者的更好选择、耐药的机制仍不清楚以及耐药后患者应如何治疗等。期待更加深入的实验研究和临床研究来解决上述问题，使患者以最小的花费取得最佳疗效，真正在基因水平上实现肺癌的个性化治疗。
